# Neutralizing Potency of Prototype and Omicron RBD mRNA Vaccines Against Omicron Variant

**DOI:** 10.3389/fimmu.2022.908478

**Published:** 2022-06-30

**Authors:** Jinkai Zang, Yannan Yin, Shiqi Xu, Weihua Qiao, Qiuyue Liu, Dimitri Lavillette, Chao Zhang, Haikun Wang, Zhong Huang

**Affiliations:** CAS Key Laboratory of Molecular Virology and Immunology, Institut Pasteur of Shanghai, Chinese Academy of Sciences, University of Chinese Academy of Sciences, Shanghai, China

**Keywords:** SARS-CoV-2, omicron variant, receptor-binding domain, mRNA vaccine, neutralizing antibody

## Abstract

The newly emerged Omicron variant of severe acute respiratory syndrome coronavirus 2 (SARS-CoV-2) contains more than 30 mutations on the spike protein, 15 of which are located within the receptor binding domain (RBD). Consequently, Omicron is able to extensively escape existing neutralizing antibodies and may therefore compromise the efficacy of current vaccines based on the original strain, highlighting the importance and urgency of developing effective vaccines against Omicron. Here we report the rapid generation and evaluation of an mRNA vaccine candidate specific to Omicron, and explore the feasibility of heterologous immunization with WT and Omicron RBD vaccines. This mRNA vaccine encodes the RBD of Omicron (designated as RBD-O) and is formulated with lipid nanoparticle. Two doses of the RBD-O mRNA vaccine efficiently induce neutralizing antibodies in mice; however, the antisera are effective only on the Omicron variant but not on the wildtype and Delta strains, indicating a narrow neutralization spectrum. It is noted that the neutralization profile of the RBD-O mRNA vaccine is opposite to that observed for the mRNA vaccine expressing the wildtype RBD (RBD-WT). Importantly, booster with RBD-O mRNA vaccine after two doses of RBD-WT mRNA vaccine can significantly increase neutralization titers against Omicron. Additionally, an obvious increase in IFN-γ, IL-2, and TNF-α-expressing RBD-specific CD4^+^ T cell responses was observed after immunization with the RBD-WT and/or RBD-O mRNA vaccine. Together, our work demonstrates the feasibility and potency of an RBD-based mRNA vaccine specific to Omicron, providing important information for further development of heterologous immunization program or bivalent/multivalent SARS-CoV-2 vaccines with broad-spectrum efficacy.

## Introduction

Since December 2019, the coronavirus disease 2019 (COVID-19) pandemic, caused by severe acute respiratory syndrome coronavirus 2 (SARS-CoV-2), has given rise to a longstanding damage throughout the world (1). With the spread of the epidemic, numerous variants of concern (VOCs) have already appeared, such as Alpha (B.1.1.7) (2, 3), Beta (B.1.351) (4), Gamma (P1) (5) and Delta (B.1.617.2) (6). Recently, a novel variant B.1.1.529 was identified (7) and declared as the fifth SARS-CoV-2 VOC, named Omicron, on 26 November 2021 by the World Health Organization (WHO). Omicron is now the dominant SARS-CoV-2 variant worldwide. Omicron is the most divergent SARS-COV-2 variant that harbors more than 30 mutations in the spike protein with 15 mutations within the receptor binding domain (RBD). Previous studies have confirmed that Omicron variant markedly reduces neutralization sensitivity to convalescent sera and the antibodies elicited by the currently approved vaccines developed based on the prototype strain (8–11). It is therefore of significant importance to develop new vaccine candidates targeting the Omicron variant as a part of the preparedness plan.

To control COVID-19 pandemic, a number of kinds of SARS-CoV-2 vaccines have been and are being developed and some have been approved or authorized for emergency use, mainly including inactivated whole-virus (12), adenovirus vector, recombinant subunit protein (13), and mRNA vaccines (14, 15). Particularly, mRNA vaccines have advantages over traditional vaccines, such as ease of design, rapid production, cell free, and the induction of robust neutralizing antibody and T cell response (15–17), leading to mRNA vaccines being the first licensed vaccines against SARS-CoV-2 infections. Indeed, mRNA vaccines have played a critical role in preventing severe COVID-19-related disease and death around the world.

In this study, we designed and prepared two mRNA vaccines encoding the RBD of SARS-CoV-2 prototype (WT) or Omicron strain, designated RBD-WT and RBD-O, respectively. We next carried out the animal immunization experiments to evaluate the immunogenicity of the two vaccines and compared the neutralization titers of SARS-CoV-2 prototype and variant strains by the vaccine immune serum samples. We found that antibodies elicited by the two mRNA vaccines exhibit distinct cross-neutralization profiles. In addition, booster with RBD-O mRNA vaccine after two doses of RBD-WT mRNA vaccine can rapidly stimulate the production of neutralizing antibodies to Omicron. Additionally, RBD-specific CD4^+^ T cell responses were observed after homologous or heterologous prime-boost immunizations.

## Materials and Methods

### Cells

HEK 293T cells were cultured in DMEM (Gibco, USA) supplemented with 10% fetal bovine serum (FBS; Gibco) at 37°C. HEK 293F cells were cultured in FreeStyle 293 expression medium (Gibco). Human ACE2-overexpressing HEK 293T cell (293T-hACE2) were generated in a previous study (18).

### Recombinant Proteins and Antibodies

RBD protein (residues R319 to S591) of SARS-CoV-2 strain Wuhan-Hu-1 (GenBank ID: MN908947.3) with a N-terminal strep-tag and C-terminal 6x His-tag was expressed in HEK 293F cells and then purified using Ni-NTA resin (Millipore).

A polyclonal antibody against HEK 293F-expressed RBD protein was generated in our previous study (18) and used for immunofluorescence staining analysis. A polyclonal antibody against *E. coli*-produced RBD protein was generated in our previous study (19) and used for western blotting analysis.

### mRNA and Lipid Nanoparticles (LNP) Production

Construction of mRNA was carried out according to a previously described protocol (20). The codon-optimized WT or Omicron RBD genes (residues R319 to N532) with N-terminal T7 promotor, 5’-untranslated region (5’-UTR: 5’-AAATAAGAGAGAAAAGAAGAGTAAGAAGAAATATAAGAGCCACC-3’), interleukin-10 (IL-10) signal sequence, C-terminal 3’-UTR (5’-TGATAATAGGCTGGAGCCTCGGTGGCCATGCTTCTTGCCCCTTGGGCCTCCCCCCAGCCCCTCCTCCCCTTCCTGCACCCGTACCCCCGTGGTCTTTGAATAAAGTCTGA-3’) and poly-A tail of about 101 base pairs (bp) were synthesized and inserted into pUC57 vector. The firefly luciferase (FLuc) reporter gene with T7 promotor, 5’-UTR, IL-10 signal, 3’-UTR and poly-A tail was cloned into pUC57 vector, used as a control. The resulting plasmids were linearized and used to produce mRNAs *in vitro* using T7 RNA Transcription Enzyme Kit (Novoprotein; catalog No. E131-01A). The resultant mRNAs were purified and then capped using vaccinia capping system (New England BioLabs, M2080S) and mRNA Cap 2´-O-Methyltransferase (New England BioLabs, M0366S) to produce the Cap I structure. The capped mRNAs were purified and stored at -80°C until use.

LNP-mRNA formulations were prepared by rapid mixing as described previously (20). Briefly, to generate LNP, D-Lin-MC3-DMA (MedChemExpress), DSPC (Avanti Polar Lipids), cholesterol (Sigma), and PEG-lipid (Avanti Polar Lipids) were solubilized in ethanol at a molar ratio of 50:10:38.5:1.5. The lipid mixture was added to 25 mM acetate buffer (pH 4.0) at a volume ratio of 1: 2 and incubated at room temperature for 2 min. Next, the lipid mixture was homogenized by extrusion through 100-nm pore-size filters (Genizer) using a lipid extruder (Genizer). For mRNA encapsulation, the capped mRNAs were solubilized in 25 mM acetate buffer and then mixed with LNP at a mRNA:lipid ratio of 0.056 mg/μmol, followed by incubation at 42°C for 30 min. Formulations were dialyzed against PBS (pH 7.4) overnight, concentrated and passed through 0.45-μm filters before storage at 4°C until use.

### Expression of the mRNA in HEK 293T Cells

*In vitro* transcribed mRNAs were transfected into HEK 293T cells in 6-well or 24-well plates using Lipofectamine 2000 Transfection Reagent (Invitrogen) following manufacturer’s instructions. At 48 h post-transfection, the culture supernatants were collected and concentrated for western blot analysis with HRP-conjugated anti-His tag antibody (Proteintech) and anti-RBD polyclonal antibody as detection antibodies. For immunofluorescence staining analysis, at 48 h post-transfection, the cells were washed with PBS, fixed with 4% paraformaldehyde and permeabilized, followed by blocking with 10% FBS and 10% BSA in PBS for 1 h. The fixed cells were incubated with mouse anti-RBD polyclonal antibody, followed by Alexa-488-conjugated anti-mouse secondary antibody (Invitrogen). Nuclei were stained with 4,6-diamidino-2-phenylindole (DAPI) in PBS. Finally, the stained cells were observed under a fluorescence microscope (Olympus).

### Animal Experiments

All the animal experiments in this study were approved by the Institutional Animal Care and Use Committee at the Institut Pasteur of Shanghai.

For bioluminescence imaging to detect *in vivo* distribution of Fluc-mRNA-LNP, female BALB/c mice aged 6-8 weeks (n = 5) were injected with 10 μg of the FLuc-mRNA-LNP *via* intramuscular route. At 6-, 12-, 24-, and 48-h after the mRNA-LNP injection, the animals were injected intraperitoneally (i.p.) with luciferase substrate (Promega). After reaction for 8 minutes, fluorescence signals were recorded by IVIS Spectrum instrument (PerkinElmer) and analyzed using Living Image software 3.0.

For animal immunization, groups of adult female BALB/c mice (8 mice per group) were intramuscularly immunized with the LNP-encapsulated RBD-WT or RBD-O mRNA vaccines (10 μg of mRNA per dose) at weeks 0 and 2. At week 6, the RBD-O mRNA-vaccinated group received a third dose of RBD-O mRNA vaccine, while the RBD-WT mRNA vaccine-immunized mice were divided to two groups, among which 4 mice were injected with the RBD-WT mRNA vaccine while the other 4 mice received with a booster shot of the RBD-O mRNA vaccine. Another group of mice (n = 5) were injected with the FLuc-mRNA-LNP formulation (10 μg of mRNA per dose), used as a control. Blood samples were collected from individual mice at weeks 2, 4, and 8 for antibody analysis.

### Enzyme-Linked Immunosorbent Assay (ELISA)

ELISA was carried out for evaluation of antigen-specific antibodies. Briefly, 96-well plates were coated with 25 ng/well of HEK 293F-expressed RBD protein at 4°C overnight. After blocking with 5% milk, individual antisera were diluted 1:100 and added to the plates, followed by incubation for 2 h at 37°C. The plates were incubated with HRP-conjugated anti-mouse IgG (Sigma) for 1 h at 37°C. After color development, absorbance at 450 nm was measured.

### Pseudovirus Neutralization Assay

Murine leukemia virus (MLV)-based pseudoviruses bearing spike proteins of WT (Wuhan-Hu-1 strain), Delta, or Omicron SARS-CoV-2 strains were produced according to our previously reported protocol (19). Mutations in the Delta S protein include T19R, del156-157, R158G, L452R, T478K, D614G, P681R and D950N. Mutations in the Omicron S protein include A67V, H69delV70del, T95I, G142D-V143del-Y144del-Y145del, N211del-L212I, ins214EPE, G339D, S371L, S373P, S375F, K417N, N440K, G446S, S477N, T478K, E484A, Q493R, G496S, Q498R, N501Y, Y505H, T547K, D614G, H655Y, N679K, P681H, N764K, D796Y, N856K, Q954H, N969K, and L981F.

All sera samples were 2-fold serially diluted and subjected to pseudovirus neutralization assay as previously described (19). The luciferase activity was measured using the luciferase assay system (Promega). The 50% neutralization titer (NT50) was defined as the highest serum dilution at which the relative luminescence units (RLUs) were reduced by 50% compared with virus control wells.

### Intracellular Cytokine Staining (ICS) and Flow Cytometry

ICS assays were performed as previously described (21), with some modifications. Briefly, spleens were collected from immunized mice at week 10, and 2 × 10^6^ splenocytes were placed in each well of 96 U-plates and stimulated with the Omicron-RBD or WT-RBD proteins at 37°C overnight. The cells with or without the PMA (Phorbol 12-myristate 13-acetate) and ionomycin stimulants were used as a positive and negative control, respectively. Cytokine secretion inhibitor (Brefeldin A) was added 3 hours before cell collection. The cells were stained with antibodies specific for mouse cell surface markers (anti-CD4/Pacific Blue and anti-CD44/Brilliant Violet 605) and then the Live/Dead^®^ Fixable Dead Cell Stain (Invitrogen). After fixation and permeabilization, the cells were stained with anti-IFN-γ/PerCP-Cy5.5, anti-IL-2/PE, anti-TNF-α/PE-Cy7 antibodies. All samples were analyzed with a Fortessa flow cytometer (BD Biosciences, USA).

### Statistics Analysis

All statistical analyses were performed using GraphPad Prism software v6.0. Statistical significance was analyzed using Student’s *t*-test.

## Results

### Design and Characterization of mRNA Vaccines Against SARS-CoV-2

**Figure 1:**
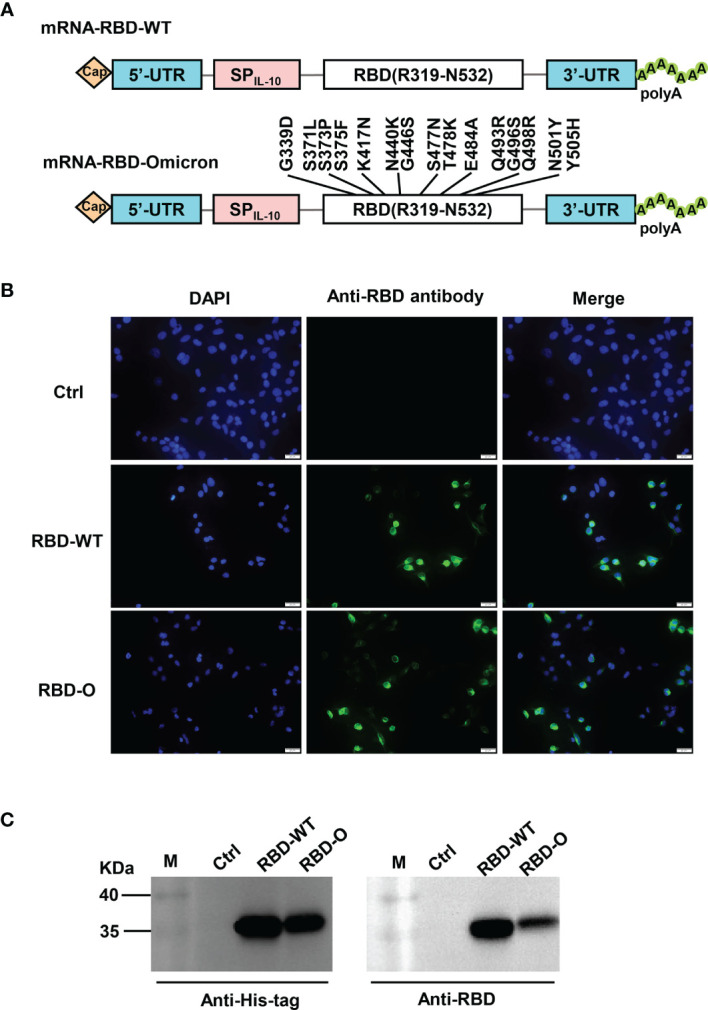
Design and characterization of LNP-encapsulated RBD mRNA vaccines against SARS-CoV-2. **(A)** Schematic diagram of RBD-WT (top panel) and RBD-Omicron (bottom panel) mRNA framework. Note that 15 mutations are located within Omicron RBD. UTR, untranslated regions; SP_IL-10_, human interleukin-10 signal peptide. **(B)** *In vitro* transcribed mRNA was transfected into HEK293T cells and the expression of RBD proteins within the cells were analyzed by immunofluorescence staining analysis with mouse anti-RBD polyclonal antibody and anti-mouse Alexa Fluor^®^ 488 secondary antibody. Ctrl, mRNA encoding luciferase. RBD-O, mRNA encoding RBD-Omicron. Overlay, merge of the blue (DAPI) and green (RBD) channels. Scale bars = 20 μm. **(C)** The culture supernatants of mRNA-transfected HEK293T cells were analyzed for RBD expression by western blotting with HRP-conjugated anti-His tag antibody and anti-RBD polyclonal antibody as detection antibodies. M, marker.

In this study, we adopted a non-replicating mRNA vaccine platform to rapidly produce and evaluate candidate vaccines against the Omicron variant. Two mRNA constructs encoding the RBD from the SARS-CoV-2 wildtype (WT) strain or from the Omicron variant were generated, designated as RBD-WT and RBD-O, respectively (**Figure 1A**). To verify the constructs, HEK293T cells were transfected with *in vitro* transcribed mRNA and subsequently analyzed for expression of the target proteins. Both RBD-WT and RBD-O mRNA-transfected cells, but not the control cells transfected with the luciferase-expressing mRNA, could be positively detected by an anti-RBD polyclonal antibody in the immunofluorescence staining assays (**Figure 1B**). For both RBD-WT and RBD-O mRNA-transfected samples, the target proteins (~35 KDa, which is close to the predicated molecular mass) could be detected in the culture medium by both anti-His-tag and anti-RBD antibodies, indicating that the target proteins were correctly expressed and secreted (**Figure 1C**). Together, the above results validate the mRNA vaccine constructs.

Next, we encapsulated the antigen-expressing mRNAs with lipid nanoparticles (LNPs) to generate mRNA vaccine stocks. To visualize the *in vivo* delivery and expression of the mRNA vaccines, a firefly luciferase (FLuc) reporter-encoding mRNA-LNP formulation prepared using the identical methods was injected into mice, followed by bioluminescence imaging at different time points. Strong bioluminescence was observed in the FLuc mRNA-LNP injected mice at 6 hours post injection, indicating robust expression of FLuc (**Supplementary Figure S1**). The bioluminescent signal then decreased gradually but remained detectable up to 48 hours post injection in some mice (**Supplementary Figure S1**). These results demonstrate that our mRNA-LNP formulations are capable of delivering mRNA into cells and expressing the target antigens *in vivo*.

### Immunization With mRNA Vaccines Elicited Neutralizing Antibodies in Mice

**Figure 2:**
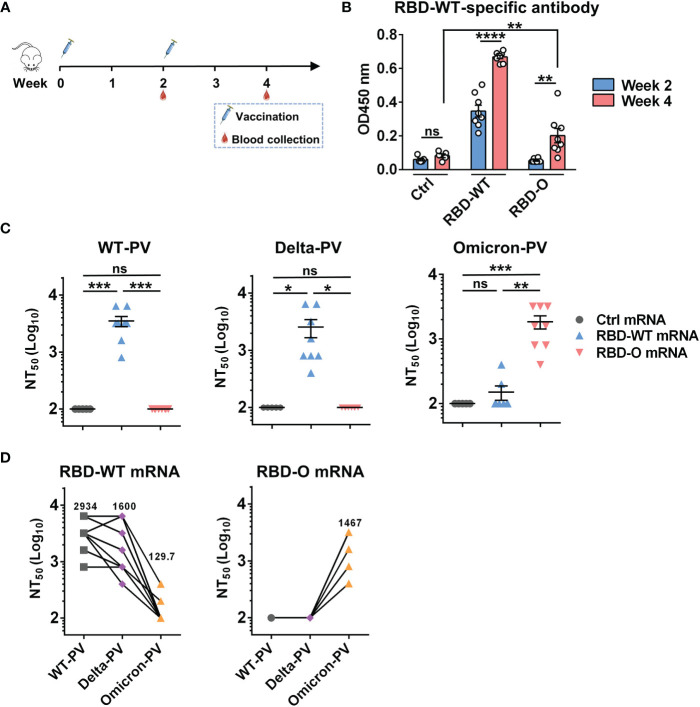
Immunization with mRNA vaccines elicited neutralizing antibodies in mice. **(A)** Mice immunization schedule. Groups of BALB/c mice were injected i.m. with 10 μg of RBD-WT mRNA (n = 8), RBD-O mRNA (n = 8), or luciferase-mRNA (Ctrl; n = 5) vaccines at weeks 0 and 2. Serum samples were collected from individual mice at weeks 2 and 4. **(B)** The week-2 and week-4 antisera were diluted 1:100 and analyzed for RBD-WT-binding activity by ELISA. **(C)** Neutralizing titers of the week-4 antisera samples from each group against WT-, Delta-, and Omicron-PV (pseudovirus). Serum samples that exhibited less than 50% neutralization at the lowest serum dilution (1:200) were assigned a NT_50_ value of 100 for statistical analysis. Each symbol represents one mouse. **(D)** Neutralizing titers of the week-4 antisera from RBD-WT mRNA group (left panel) and RBD-O mRNA group (right panel) against SARS-CoV-2 PV. The geometric mean of NT50 values was shown. For panels **(B, C)**, data are presented as mean ± SEM. *p* values were analyzed with unpaired *t*-test and indicated as follows: ns, not significant; *p < 0.05; **p < 0.01; ***p < 0.001; ****p < 0.0001.

We then performed mouse immunization experiments to assess the immunogenicity and efficacy of the mRNA vaccine candidates. Two groups of female BALB/c mice (8 animals/group) were intramuscularly (i.m.) immunized with the RBD-WT or the RBD-O mRNA vaccine, respectively, at weeks 0 and 2 (**Figure 2A**). Another group of mice (5 animals/group) were injected with the FLuc-expressing mRNA-LNP formulation, serving as the control in the experiment. Serum samples were collected from individual mice at weeks 2 and 4 for antibody titration and functional analysis (**Figure 2A**).

The antisera were first evaluated for the presence of antigen-specific antibodies by ELISA with mammalian cell-produced recombinant RBD-WT as the capture antigen. All antisera were diluted 1:100 and then subjected to ELISA. The results were shown in **Figure 2B**. The sera from the control (Fluc mRNA) group produced only background level of OD450 nm reading. In contrast, strong RBD-WT binding activity was observed for the RBD-WT mRNA vaccine sera collected at week-2 and the binding signal increased significantly at week-4, demonstrating the elicitation of antigen-specific binding antibodies by the mRNA vaccine. In addition, none of the week-2 antisera from the RBD-O mRNA vaccine group exhibited significant binding activity to RBD-WT, however, low levels of RBD-WT binding were detected in some of the week-4 antisera, suggesting that the RBD-O mRNA vaccine is immunogenic despite its ability to induce cross-reactive antibodies is limited. We also produced mammalian cell-expressed recombinant Omicron RBD protein. However, this protein exhibited aberrant binding orientation/coating efficiency on ELISA plate (**data not shown**) probably due to its surface property change resulting from the large number of amino acid substitution, and therefore was not used as the coating antigen in ELISA-based antibody measurement.

The week-4 antisera were assessed for their neutralization potency and breadth against a small panel of SARS-CoV-2 pseudovirus (PV), including the WT-PV, Delta-PV, and Omicron-PV (**Figure 2C**). As expected, none of the antisera from the control (FLuc) group displayed neutralization towards any of the three PVs. Note that the serum samples unable to produce ≥50% neutralization when diluted 1:200 (the lowest serum dilution tested in the neutralization assays) were assigned a titer of 100 for geometric mean titer (GMT) computation. In contrast, all antisera from the RBD-WT mRNA vaccine group could potently neutralize the WT-PV with titers ranging from 800 to 6400 (GMT=2934) and also neutralize the Delta-PV with a slightly decreased efficiency (GMT=1600), however, only two out of the eight antisera marginally neutralized the Omicron-PV with titers being 200 and 400, respectively. For the RBD-O mRNA vaccine group, all antisera efficiently neutralized the Omicron-PV with a neutralizing GMT of 1467, but none of them exhibited cross-neutralization against the WT-PV and Delta-PV (**Figure 2C**).

To better characterize the antisera’s cross-neutralization capacity, the NT50 titers of individual mouse sera against the three PVs were directly compared (**Figure 2D**). For individual RBD-WT mRNA vaccine sera, their neutralization potency appeared not significantly affected by the Delta variant (less than 2-fold change in neutralizing GMT) but decreased drastically or even completely lost towards the Omicron variant. Conversely, all of the RBD-O mRNA vaccine sera were able to efficiently neutralize the Omicron variant with titers ranging from 400 to 3200, however, none of them displayed neutralization effects on the WT strain or the Delta variant.

### Heterologous Booster Immunization Programs Increase Neutralization Titers and Breadth

**Figure 3:**
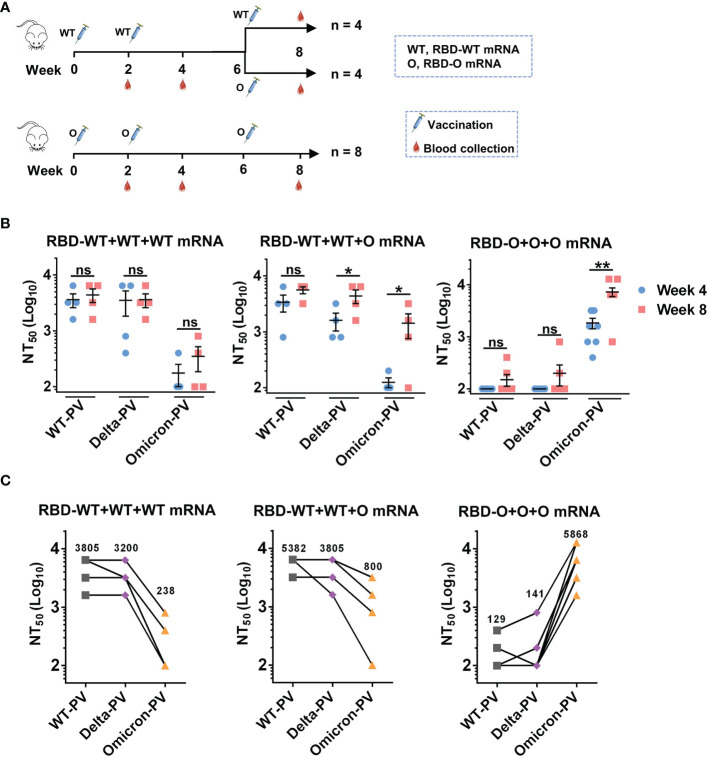
The influence of homologous and heterologous booster immunization programs on serum neutralization titers and breadth. **(A)** Mice booster immunization schedule. The original RBD-WT mRNA vaccine group was divided into two (n = 4 mice per group): group I (the RBD-WT+WT+WT mRNA group) mice were boosted with the RBD-WT mRNA vaccine, and group II (the RBD-WT+WT+O mRNA group) mice with the RBD-O mRNA vaccine. The original RBD-O mRNA vaccine group (re-named the RBD-O+O+O mRNA group) received a third dose of the RBD-O mRNA vaccine. Serum samples were collected at week 8. **(B)** Neutralizing titers of the week-4 and week-8 antisera samples from each group against WT-, Delta-, and Omicron-PV (pseudovirus). If serum samples show less than 50% neutralization at the 1:200 dilution, a NT_50_ value of 100 was assigned for statistical analysis. ns, not significant; *p < 0.05; **p < 0.01. **(C)** Neutralizing titers of the week-8 antisera from the three vaccine groups against SARS-CoV-2 PVs. The geometric mean of NT50 values was shown.

Since two doses of the RBD-WT mRNA vaccine failed to induce anti-Omicron neutralizing antibody responses (**Figures 2C, D** **)**, we wanted to test whether a third dose of the heterologous RBD-O mRNA vaccine could enhance cross-neutralizing antibodies to the currently circulating Omicron variant. To this end, the RBD-WT mRNA vaccine group (n = 8 mice) was randomly divided into two groups (n = 4 per group) (**Figure 3A**). The first group (designated as the RBD-WT+WT+WT mRNA group) received a booster of the homologous RBD-WT mRNA vaccine at week 6, while the second group (designated as the RBD-WT+WT+O mRNA group) received a third dose of the heterologous RBD-O mRNA vaccine at week 6. The original RBD-O mRNA vaccine group (n = 8 mice) continued to receive a third dose of RBD-O mRNA vaccine at week 6 and was referred to as the RBD-O+O+O mRNA group (**Figure 3A**). Next, antisera samples were collected at week 8 (two weeks after the third dose) and used for neutralization analysis (**Figures 3B, C**). For the RBD-WT+WT+WT mRNA group, the third dose of RBD-WT mRNA vaccine increased neutralizing antibody levels against WT-PV and Delta-PV with GMTs of 3805 and 3200, respectively, compared to their levels after the second dose (GMTs = 3200 for WT-PV, 1903 for Delta-PV). However, after the third dose, only two mice in the RBD-WT+WT+WT mRNA group developed anti-Omicron neutralizing antibody responses, and the neutralizing GMTs against Omicron-PV were still low (GMT = 238). For the RBD-WT+WT+O mRNA group, booster with the heterologous RBD-O mRNA vaccine was also able to increase neutralization titers against WT-PV and Delta-PV with GMTs of 5382 and 3805, respectively, relative to their levels after the second dose (GMTs = 2690 for WT-PV, 1345 for Delta-PV). Notably, for the week-8 sera from the RBD-WT+WT+O mRNA group, neutralizing GMTs against Omicron-PV increased 6.7-fold relative to their levels at week 4 (GMT of 800 versus 119). Therefore, booster with the heterologous RBD-O mRNA vaccine could elicit moderate neutralization of Omicron variant. For the RBD-O+O+O mRNA group, the third vaccination resulted in significantly higher neutralization titers (GMT = 5868) against Omicron-PV, as compared with the titers (GMT = 1467) after the second immunization. Following the third dose, only 2 out of 8 mice in the RBD-O+O+O mRNA group developed neutralizing antibody response against WT-PV and Delta-PV with GMTs less than 150. Thus, three doses of RBD-O mRNA vaccine could not elicit strong anti-WT/anti-Delta neutralizing antibody responses.

### mRNA Vaccines Elicit SARS-CoV-2-Specific T Cell Immune Response in Mice

**Figure 4:**
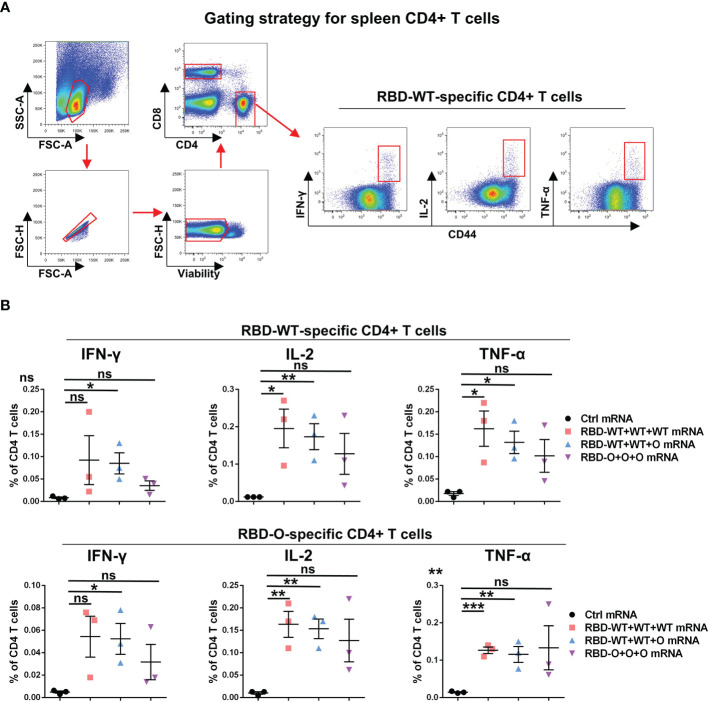
RBD-WT- and RBD-O-specific CD4 T-cell intracellular cytokine staining (ICS) assays. The splenocytes isolated from control or vaccinated individual mice were stimulated with Omicron-RBD or WT-RBD proteins overnight, and the expression of the intracellular cytokines IFN-γ, IL-2, and TNF-α in CD4 T cells was analyzed by flow cytometry. **(A)** Representative gating strategy for the identification of antigen-specific CD4 T cells within the mouse spleen. **(B)** Frequencies of RBD-WT- and RBD-O-specific CD4 T cells in spleen were measured by intracellular cytokine staining. Each symbol represents one mouse. Data are presented as mean ± SEM. *p* values were analyzed with unpaired t-test and indicated as follows: ns, not significant; *p < 0.05; **p < 0.01; ***p < 0.001.

In addition to humoral immunity, cellular immunity also plays the vital role of countering infection (22, 23). To evaluate the T cellular immune responses generated by the RBD mRNA vaccines, splenic lymphocytes from control and vaccinated individual mice were harvested at week 10 and stimulated overnight with the recombinant RBD-WT or RBD-O protein. The expression of canonical Th1 cytokines, including interferon (IFN)-γ, tumor necrosis factor (TNF)-α, and interleukin (IL)-2, in CD4^+^ T cells was analyzed by intracellular cytokine staining (ICS) assay (**Figure 4A**). As shown in **Figure 4B**, after RBD-WT antigen stimulation, no cytokine production in CD4^+^ T cells were detected in the control mRNA group. In contrast, higher proportions of IFN-γ, IL-2, and TNF-α positive CD4^+^ T cells were detected in each tested mouse of the RBD-WT+WT+WT, RBD-WT+WT+O, and RBD-O+O+O mRNA vaccine groups. Similarly, after RBD-O antigen stimulation, no detectable IFN-γ, IL-2, and TNF-α positive CD4^+^ T cells were observed in any of the control mice, while higher frequencies of cytokines positive CD4^+^ T cells were observed in each mRNA vaccine-immunized mouse. It is worth noting that cross-reactive T cell responses were observed in the three vaccine groups when splenocytes were stimulated with the heterologous RBD antigen, suggesting several T cell epitopes are shared by different RBD antigens. Taken together, these data demonstrate that the mRNA vaccines can efficiently trigger RBD-specific T cellular immune responses.

## Discussion

The present study compares side-by-side two mRNA-based SARS-CoV-2 vaccine candidates that express the RBD of the WT strain or the Omicron variant, respectively, for their immunogenicity and neutralization capacity. It was found that both of the mRNA vaccines are highly immunogenic, able to produce relatively high titers of neutralizing antibodies against the homologous strains. However, antibodies elicited by the two vaccines display distinct cross-neutralization profiles. Specifically, the RBD-WT mRNA vaccine sera exhibited slightly decreased (1.83-fold) neutralization potency on the Delta variant but were strikingly escaped by the Omicron variant (more than 22.6-fold reduction in neutralizing GMT), in consistent with the results obtained with human vaccinee sera (8, 9, 24). Significantly, we found that the RBD-O mRNA vaccine sera potently neutralized the Omicron variant but failed to cross-neutralize the WT or Delta strains under the testing conditions used in our study, indicating a narrow neutralization spectrum for this vaccine. Since the currently approved COVID-19 vaccines are based on the original WT SARS-CoV-2 strains and are not effective against the dominant circulating virus Omicron, we carried out a novel immunization program in this study, two doses of WT RBD mRNA plus a third booster dose of Omicron RBD mRNA. We found that this program could significantly increase neutralization titers against Omicron. Based on the above results, we propose that a heterologous immunization program or bivalent/multivalent vaccine formulation containing antigen components (such as RBD) derived from the Omicron variant and the WT strain (or an earlier variant such as Delta) should be developed in order to achieve broad protection against diverse SARS-CoV-2 strains/variants, especially Omicron and Delta. In addition, the effect of the homologous booster vaccination on neutralization antibody levels was also investigated in this study. We found that three doses of RBD-O mRNA vaccine did not significantly increase the neutralization titers to any tested PV, compared to two doses of RBD-WT mRNA vaccine. However, three doses of RBD-O mRNA vaccine could significantly improve the neutralization of Omicron-PV but not WT-PV and Delta-PV.

S and RBD proteins are two major targets for vaccine development. In this study, mRNA vaccines against Omicron variant were developed by using the RBD target. As mentioned above, the RBD-O mRNA vaccine encoding RBD alone was shown to be very effective at neutralizing Omicron variant. In addition, choosing the RBD but not S protein as the target is believed to minimize the potential risks of antibody-dependent enhancement (ADE) in the SARS-CoV-2 infections (25). It should be pointed out that the biggest shortcoming of the RBD-O mRNA vaccine is the narrow serum neutralization breadth, probably because that Omicron mutations impact most antibody epitopes in the RBD region (26–28).

The currently approved mRNA vaccines (e.g. Moderna mRNA-1273) encode the full-length S protein of the original SARS-CoV-2 strain. A recent study reported that after immunization with two doses of mRNA-1273 vaccine, neutralization titers of recipients’ sera against Omicron were 35 times lower than those against D614G variant. However, after the third immunization of mRNA-1273 vaccine, neutralization titers against Omicron were 20 times higher than those evaluated after the second immunization (29). In our study, we prepared two experimental mRNA vaccines encoding only the RBD region of the prototype or Omicron strain, respectively. We found that after two doses of RBD-WT mRNA vaccine, the sera from only 25% (two out of eight) immunized mice can weakly neutralize Omicron with titers being 200 and 400, respectively (**Figure 2C**), and the third dose does not significantly improve the poor cross-neutralization activity of Omicron (**Figure 3B**). By contrast, 100% mice treated with two doses of RBD-O mRNA vaccine developed strong anti-Omicron neutralizing antibody responses with a GMT of 1467 (**Figures 2C, D**), and the neutralizing titer (GMT) for Omicron further increased to 5868 after the third dose (**Figures 3B, C**).

Previous reports have demonstrated that CD4^+^ T cells, especially follicular helper T cells (Tfh cells), can help B cells to produce stronger antibody responses against many viral pathogens (30). The close connection between CD4^+^ T cells and antibody production in COVID-19 convalescent patients has been demonstrated (31). A clinical trial with COVID-19 vaccine BNT162b1 has also shown that the strength of the vaccine-induced RBD-specific CD4^+^ T cell responses is positively correlated with neutralizing antibody titers against SARS-CoV-2 (17). In addition to B cell helper functions, CD4^+^ T cells can contribute directly to viral clearance by secreting cytokines with antiviral activities (30). As a consequence, the COVID-19 vaccines-induced CD4^+^ T cell responses may play an important role in protection against hospitalization or death. In this study, we found that RBD-specific CD4^+^ T cell responses are readily detected after immunization with the RBD-WT and/or RBD-O mRNA vaccines (**Figure 4**). Interestingly, unlike the poor cross-neutralization activity of antibodies indued by RBD-O mRNA vaccine, Omicron RBD specific CD4^+^ T cells respond well to WT RBD protein stimulation, suggesting conserved CD4^+^ T epitopes existing in the RBD region.

In short, our current work demonstrates the feasibility and potency of an RBD-based mRNA vaccine specific for the SARS-CoV-2 Omicron variant, providing important information for further development of bivalent or multivalent SARS-CoV-2 vaccines with broad-spectrum efficacy.

## Data Availability Statement

The raw data supporting the conclusions of this article will be made available by the authors, without undue reservation.

## Ethics Statement

The animal study was reviewed and approved by Institutional Animal Care and Use Committee at the Institut Pasteur of Shanghai.

## Author Contributions

ZH and HW conceived and designed the experiments; JZ, CZ, YY, and QL participated in multiple experiments; ZH, HW, JZ, CZ, and YY analyzed the data; ZH, HW, JZ, CZ, and YY wrote the manuscript; ZH and HW provided the final approval of the paper. All authors contributed to the article and approved the submitted version.

## Funding

This work was supported by grants from the Chinese Academy of Sciences (XDB29040300, XDB29030103) and from the Ministry of Science and Technology of China (2020YFC0845900), National Key R&D Program of China (2021YFA1301402), and Shanghai Municipal Science and Technology Major Project (ZD2021CY001). This project is also part of the European Union’s Horizon 2020 research and innovation program under grant agreement No.101003589. CZ is supported in part by Youth Innovation Promotion Association of the Chinese Academy of Sciences (CAS) and Shanghai Rising-Star Program (21QA1410000).

## Conflict of Interest

The authors declare that the research was conducted in the absence of any commercial or financial relationships that could be construed as a potential conflict of interest.

## Publisher’s Note

All claims expressed in this article are solely those of the authors and do not necessarily represent those of their affiliated organizations, or those of the publisher, the editors and the reviewers. Any product that may be evaluated in this article, or claim that may be made by its manufacturer, is not guaranteed or endorsed by the publisher.
