# Supplementary material for: Neutralizing Potency of Prototype and Omicron RBD mRNA Vaccines Against Omicron Variant
Source: Front Immunol. 2022 Jun 30;13:908478. doi: 10.3389/fimmu.2022.908478 (PMC9280631; doi:10.3389/fimmu.2022.908478)
Supplement: Supplementary file 1 [file DataSheet_1.pdf]

Supplementary Information

Jinkai Zang[#], Yannan Yin [#], Shiqi Xu, Weihua Qiao, Qiuyue Liu, Dimitri Lavillette, Chao Zhang*, Haikun Wang*, Zhong Huang *

[1] CAS Key Laboratory of Molecular Virology & Immunology, Institut Pasteur of Shanghai, Chinese Academy of Sciences, University of Chinese Academy of Sciences, Shanghai, China

[#] These authors contributed equally.

* Corresponding author: Zhong Huang (huangzhong@ips.ac.cn), Haikun Wang (hkwang@ips.ac.cn) or Chao Zhang (chaozhang@ips.ac.cn)

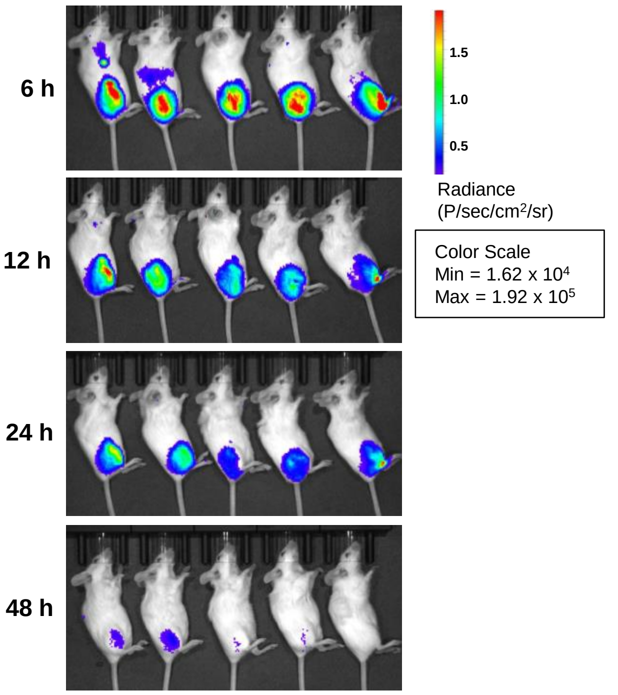

Supplementary Fig. S1 Characterization of the mRNA-LNP expression in vivo. Mice were injected with the FLuc-mRNA-LNP formulation and then subjected to bioluminescence imaging at different time points.
